# Preferences, beliefs, and attitudes about oral fluid and blood-based HIV self-testing among truck drivers in Kenya choosing not to test for HIV

**DOI:** 10.3389/fpubh.2022.911932

**Published:** 2022-11-09

**Authors:** Joanne E. Mantell, Aleya Khalifa, Stephanie N. Christian, Matthew L. Romo, Eva Mwai, Gavin George, Michael Strauss, Kaymarlin Govender, Elizabeth A. Kelvin

**Affiliations:** ^1^Department of Psychiatry, HIV Center for Clinical and Behavioral Studies, New York State Psychiatric Institute, Columbia University Irving Medical Center, New York, NY, United States; ^2^ICAP at Columbia University, New York, NY, United States; ^3^Department of Epidemiology, Mailman School of Public Health, Columbia University, New York, NY, United States; ^4^Department of Behavioral and Community Health Sciences, University of Pittsburgh School of Public Health, Pittsburgh, PA, United States; ^5^Department of Epidemiology and Biostatistics and Institute for Implementation Science in Population Health, CUNY Graduate School of Public Health and Health Policy, City University of New York, New York, NY, United States; ^6^The North Star Alliance, Nairobi, Kenya; ^7^Health Economics and HIV/AIDS Research Division (HEARD), University of KwaZulu-Natal, Durban, South Africa; ^8^Division of Social Medicine and Global Health, Lund University, Lund, Sweden

**Keywords:** HIV, HIV testing, HIV self-testing (HIVST), HIV/AIDS (acquired immunodeficiency syndrome), implementation science, truck driver, Kenya

## Abstract

**Introduction:**

Clinical trials in sub-Saharan Africa support that HIV self-testing (HIVST) can increase testing rates in difficult-to-reach populations. However, trials mostly evaluate oral fluid HIVST only. We describe preferences for oral fluid vs. blood-based HIVST to elucidate prior trial results and inform testing programs.

**Methods:**

Participants were recruited from a HIVST randomized controlled trial in Nakuru County, Kenya, which aimed to test the effect of choice between oral HIVST and facility-based testing compared to standard-of-care on HIV testing among truck drivers. We conducted in-depth interviews (IDIs) with purposively sampled trial participants who declined HIV testing at baseline or who were offered access to oral fluid HIVST and chose not to pick up the kit during follow-up. IDIs were conducted with all consenting participants. We first describe IDI participants compared to the other study participants, assessing the statistical significance of differences in characteristics between the two samples and then describe preferences, beliefs, and attitudes about HIVST biospecimen type expressed in the IDIs.

**Results:**

The final sample consisted of 16 men who refused HIV testing at baseline and 8 men who did not test during follow-up. All IDI participants had tested prior to study participation; mean number of years since last HIV test was 1.55, vs. 0.98 among non-IDI participants (*p* = 0.093). Of the 14 participants who answered the question about preferred type of HIVST, nine preferred blood-based HIVST, and five, oral HIVST. Preference varied by study arm with four of five participants who answered this question in the Choice arm and five of nine in the SOC arm preferring blood-based HIVST. Six key themes characterized truckers' views about test type: (1) Rapidity of return of test results. (2) Pain and fear associated with finger prick. (3) Ease of use. (4) Trust in test results; (5) fear of infection by contamination; and (6) Concerns about HIVST kit storage and disposal.

**Conclusion:**

We found no general pattern in the themes for preference for oral or blood-based HIVST, but if blood-based HIVST had been offered, some participants in the Choice arm might have chosen to self-test. Offering choices for HIVST could increase testing uptake.

## Introduction

HIV testing services in east and southern Africa have been scaled up over the last decade; however, HIV testing remains sub-optimal for certain groups, such as men. According to UNAIDS, men and boys living with HIV in the region are 20% less likely than women and girls living with HIV to know their status (1). In Kenya, 94% of women living with HIV compared to 88% of men living with HIV knew their status in 2020 (2). These statistics translate into 61,000 Kenyan men living with HIV remaining undiagnosed compared to 53,000 Kenyan women, despite a higher prevalence among women (5.8%) than men (3.2%) (3).

Male migrant workers are at an even higher risk of not knowing their HIV status. In Kenya, long-distance truck drivers who drive along major transport corridors are an important priority population because, like many mobile populations, they experience increased risk of both HIV acquisition and onward transmission (4–6). Their mobility has been identified as a key structural driver of risk of HIV infection that may be fueled by alcohol and drug use, inconsistent condom use (7), concurrent and multiple sexual partnerships (8), and use of commercial sex services during their road trips while away from regular partners/wives (9–12). Furthermore, mobile populations face various challenges in accessing continuous and quality HIV prevention services (13–15). More specifically, truck drivers experience barriers to facility-based HIV testing due to their strict time schedule, lack of personal transport, and variation in their eligibility for services as they move across geographic borders. Thus, truck drivers need flexible HIV testing services such as HIV self-testing (HIVST) that empower them to manage their own health while on the move. HIVST can also reduce the stigma associated with facility-based testing and provide real-time feedback for populations unable to access a clinic.

HIV self-testing is a promising approach to increasing testing uptake, particularly for mobile populations like long-distance truck drivers. However, little is known about the acceptability and preferences regarding the different forms of HIVST among this important group. Studies suggest that some people may place greater trust in blood over oral fluid for identifying HIV infection (16), as evidenced in interviews with clinic clients in South Africa (17) as well as among men who have sex with men (MSM) in South Africa where there was a 2:1 preference for the blood-based kit compared to oral HIVST (16). In our in-depth interviews among truck drivers in Kenya who refused HIV testing, a fair number placed greater trust in the blood test but also expressed mistrust that HIV can be detected in oral fluid (18). However, preference was stronger for using an oral fluid HIVST than a blood-based HIVST in a study among MSM in New York City, but the likelihood of using a blood-based test increased if the test could also detect other STIs (16, 19). Information about preferences, including which testing and distribution options will maximize truckers' HIV testing rates, as well as the reasoning behind those preferences and if they are related to mistrust in healthcare services or providers or misinformation, is key to developing HIVST programs that maximize their potential to increase the number of people who know their HIV status.

Three randomized controlled trials (RCTs) among truckers and female sex workers in Kenya conducted by our team found that offering choices between the standard provider-administered rapid finger-prick HIV test and an oral HIVST increased testing uptake over the offer of only the standard option [odds ratios [OR] ranged from 2.8–3.7 for truckers (20, 21) and 2.9 among sex workers (22)].The PopART cluster-randomized trial in Zambia found similar results when offering the choice of oral HIVST in addition to lay counselor-administered finger prick testing door-to-door overall (Aor = 1.30) and among men (Aor = 1.42) (23). Furthermore, in these studies, there was variation in the HIV test selected among those who accepted testing. In the Kenya studies, while the majority of those who tested chose the HIVST, a sizeable proportion (40.3–26.9%) selected the standard provider-administered blood test; among those who chose to self-test, there was variation in the three Kenya studies and in the Zambia study in choosing between provider-supervised HIVST or taking an HIVST kit for home use, with the majority opting for supervised HIVST. However, a key gap that remains in the literature about HIV testing preferences is whether the choice to self-test in previous studies was driven primarily by preferences regarding who administers the test (self or provider), or by the biological sample used in the test (blood or oral).

HIVST is included in Kenya's 2015 national HIV testing guidelines which supports both oral and blood-based tests (24). In May 2017, the Kenyan government approved three HIVST kits: the OraQuick HIV Self-Test (OraSure Technologies, USA) which uses an oral fluid sample, the INSTI HIV Self-Test (Pouch) (INSTI, bioLytical Laboratories, Canada), and the Atomo HIV Self-Test (Atomo Diagnostics, Australia); both of the two latter tests use a whole blood sample (25, 26). In 2019 alone, 400,000 HIVST kits had been distributed in the country (3). Currently, the OraQuick HIVST is available in limited supply in government health facilities and PEPFAR-supported programs, whereas both blood-based and oral HIVST kits are sold in select pharmacies (27). To date, most research on HIVST in sub-Saharan Africa (SSA) has focused on only the oral HIVST option, to the exclusion of other types of self-tests (28), and with the increasing availability of HIVST in the region, it is crucial to identify key factors that may increase their uptake.

Therefore, in this study we explore preferences, beliefs, and attitudes about HIVST biospecimen characteristics and participants' reports of the reasoning behind these preferences using data from in-depth interviews (IDIs) among truck drivers in Kenya who refused the offer of HIV testing in an RCT evaluating the impact of adding HIVST to clinic services. This paper differs from our published paper on truck drivers' views of the intrapersonal, interpersonal, institutional and community, and policy-level facilitators and barriers to HIVST among the same sample used in this paper (18). Using insights from those who chose not to test for HIV when offered only the standard provider-administered finger-prick test or when offered HIVST as an additional choice, we expect to better understand if different HIVST characteristics, such as a blood-based HIVST, might have been more appealing and likely to be accepted and the thinking behind these preferences. By exploring the reasons why people refused HIV testing even when offered choices, we aim to inform the development of HIVST programs to ensure the availability of preferred HIVST kits and address any mistrust or misinformation that might be behind the decisions not to access HIV testing even when multiple options are made available.

## Materials and methods

### Study setting

This study was conducted in two of the North Star Alliance clinics along the northern transport corridor in Nakuru County, Kenya, from October-December 2015 where approximately 30% of the 400 clients served weekly are truck drivers (20). Clients are offered HIV testing at every clinic visit and about 60% of truck driver clients accept testing, of whom about 1.5% test HIV-positive (20). The North Star Alliance is a non-governmental organization that delivers health services in SSA to difficult-to-access populations, including truck drivers, sex workers and the local communities with which they interact, at roadside well-ness clinics. The most recent statistics from North Star Alliance show that 91 clinics had been established by the North Star Alliance or were using North Star Alliance operational tools in 20 countries. Eight of these roadside well-ness clinics are in Kenya. The clinics are open at hours that are convenient to truck drivers and offer a range of prevention and treatment services, including HIV counseling and testing and, in some clinics, ART for treatment and prevention, primary healthcare, sexually transmitted infection screening and treatment, tuberculosis screening and treatment, behavior change communication, and laboratory services (29).

### Overview of parent RCT study design

Truck drivers who came to the participating clinics during recruitment were informed of the study by Fieldworkers, and if interested, were screened for study eligibility. Eligibility criteria for inclusion in the trial were: (1) At least 18 years old. (2) Male. (3) Work as a truck driver. (4) reside in Kenya. (5) Speak English or Kiswahili. (6) Self-report HIV-negative or unknown HIV status. (7) Able to sign the consent form, and (8) willing to receive payment for a study participation incentive *via* MPesa (a mobile phone-based money transfer system). Truck drivers who consented to study participation were randomized to be offered (1) a provider-administered blood-based fingerstick HIV test (Colloidal Gold), which was the standard of care (SOC arm), or (2) a choice of either the provider-administered rapid blood-based test or a self-administered rapid oral HIV test (OraQuick In-Home HIV Test) with provider supervision in the clinic (Choice arm). Participants in the Choice arm who refused HIV testing in the clinic were offered a test kit for home use, supported by phone-based posttest counseling. All interviews were conducted in Kiswahili, English, or a combination of the two languages, depending upon a participant's preference. A detailed description of the methods for the RCT can be found elsewhere (20). To mitigate potential non-compliance or contamination bias, participants were blinded to the study research question and to randomization to study arms offering different HIV testing options. Participants in the SOC arm were not counseled on oral HIVST kits because they were not publicly available at the time.

### Sampling

Twenty-four participants were purposively sampled from study clinics for the IDIs, including 16 who refused HIV testing at baseline (11 in the SOC and 5 in the Choice arm) and eight participants in the Choice arm who did not self-test during follow-up when they could pick-up an HIVST kit at any of the eight North Star Alliance clinics in Kenya in addition to accessing SOC testing. All of the eight Choice arm IDI participants selected because they did not test during follow-up had tested at baseline (four *via* provider-supervised oral HIVST and four *via* provider-administered blood test). All participants recruited for the IDIs agreed to participate, and none dropped out of the study.

Through the IDIs, we aimed to understand preferences, barriers and facilitators to HIV testing in general, and HIVST specifically. We focused on HIV test refusers because we sought to understand why people refuse HIV testing when offered different testing options and what might be done in the future to address the barriers non-testers experience. Our sample size estimate of eight participants per group was based on our expectation of reaching thematic saturation with our relatively homogenous sample of male truck drivers (30).

### Recruitment and procedures

Fieldworkers invited RCT participants who met eligibility criteria to participate in one IDI after they refused HIV testing at baseline and completed the final baseline quantitative interview or after completing the 6-month follow-up interview in which they reported not having tested for HIV during follow-up. Recruitment continued until we achieved our sample size. Recognizing that some participants might not have the time to complete the baseline IDI at the clinic directly following the second quantitative interview about refusal of HIV testing, participants were offered the option to schedule these qualitative interviews at another time, either in-person or by phone. Consequently, these interviews were conducted 3–6 months later. IDIs with men who did not test during follow-up were conducted between one and three months following their quantitative follow-up interview.

Interviews lasted between 45 min and 1 h and participants received 450 Kenyan Shillings (Ksh) (~ US$4.45 based on average exchange rate in 2016) for completing the interview. Interviews were conducted by four trained male and female bilingual Kiswahili-English Fieldworkers in-person (*n* = 16) or by phone (*n* = 8) in a private space at the clinic. Three of the interviewers had Bachelors' degrees (two in Counseling Psychology and one in Clinical Medicine and Surgery); the fourth interviewer had a diploma in Community Development. Interviewers were experienced in working with the target population and were trained in qualitative interviewing by the study investigators, including enhancement of skill sets (e.g., listening, reflecting, summarizing), reflexivity and self-evaluation of their positionality in qualitative inquiry, and research ethics (31). Interviews were digitally audio-recorded, de-identified, transcribed, and if in Kiswahili, translated into English. To ensure accuracy of the translation, a bilingual Kiswahili-English speaking member of the research team reviewed the transcripts for quality and fidelity prior to coding and analysis.

### Measures

We developed a semi-structured interview guide to elicit participant reports about barriers and facilitators to HIV testing in general and HIVST specifically, including beliefs and attitudes about oral and blood-based finger prick HIVST, general health beliefs, and HIV risk perception. Following general questions about HIV testing, we introduced a module of questions about self-testing with the following statement: “There are several different ways that people can self-test for HIV. First, I would like to get your thoughts about HIV self-testing in general and then talk with you about specific self-testing methods.” The following questions were then asked regarding both oral and blood-based finger prick HIVST: “What do you think of this method of testing?” “What level of confidence will you have to use this method?” “Why do you feel you have this confidence?” “If the kit is made available, would you use it?” “What are the chances of you recommending this method of testing to your friends and family?” and “How much would you be willing to pay for the test kit?” The IDI guide was not piloted as the interview domains were tested in the baseline quantitative interview.

### Analytic strategy

We characterized IDI participants and compared them to the remainder of the sample using their baseline interview data. The statistical significance of differences in categorical variables between the two samples was assessed with a chi-square test and differences in numeric variables with a Wilcoxon rank-sum (Mann-Whitney) test. Fisher's exact test was used where any cell count was <5.

The analytic process for analyzing the IDI data was iterative. Framework Analysis, a systematic deductive approach, to generate themes based on topics from the interview guide (32), was initially used for qualitative data analysis, followed by an inductive process in which the researchers identified emergent themes or patterns of meaning in the data (33). All coding and data analyses were conducted on the English language transcripts. First, three research staff (JEM, SC, and MR) in the US developed a coding scheme independently, based on their reading of the same three transcripts. Additional transcripts were then assigned for the research team to apply the codes. Nine interviews were double-coded to ensure that the researchers were segmenting the text and applying the codes in the same way. The coding team engaged in critical reflexivity (31, 34), discussed coding discrepancies, and achieved consensus on codes using the “negotiated agreement” approach to ensure consistent code interpretation and application to the text (35, 36). Thematic saturation was achieved when no new codes emerged. Primary codes based on the interview questions and secondary codes that emerged from the data were used to identify themes and relationships between themes by intervention condition. The coders also checked with the Kenyan interviewers to address questions they had about translation, linguistic nuances and cultural meanings, which in turn, enhanced interpretation of participants' narratives and inter-rater reliability. To determine inter-rater reliability, we randomly selected and compared 10 codes (out of 64) from 122 randomly selected excerpts in the nine double-coded interviews. Overall, interrater reliability of the codebook application was substantial (Pooled Cohen's k = 0.70) (18, 37, 38). To organize the results, preferences were characterized by statements of “would use” or a positive view of type of biospecimen. Transcripts were uploaded into Dedoose Version 6.2.21, a software for systematic data management of qualitative and mixed-methods data (SocioCultural Research Consultants, LLC, Los Angeles, CA; www.dedoose.com).

### Ethical considerations

This study protocol and procedures were approved by the institutional review boards/research ethics committees of The City University of New York, the Kenya Medical Research Institute, and the University of KwaZulu-Natal. Written informed consent was obtained from all participants interviewed in-person) and oral consent when participants were interviewed over the phone prior to commencement of the IDI. All research staff completed training in research ethics. The study reporting adhered to the Consolidated Criteria for Reporting Qualitative Research (COREQ) 32-item checklist to demonstrate transparency of reporting and the study's methodological strength (39). Study participants were not involved in development of the IDI guide or review of the transcripts.

## Results

### Description of the sample

Table 1 describes the characteristics of the sample. The 24 IDI participants had a median age of 36.5 years (Inter-Quartile Range (IQR): 28.0–44.5), which was similar to the median age for the remaining RCT participants (median = 36.0; IQR = 32.0–41.0, *p* = 0.846). The proportions of IDI participants who were high school graduates and earned the highest income of 24,000–55,000 Ksh (US$237.60–US$544.50 based on the average exchange rate in 2016) from trucking were similar to that of the remaining RCT participants (*p* = 0.851 and *p* = 0.513, respectively), with 37.5% of IDI participants being a high school graduate and 72.7% earning the highest income category. The only variables on which IDI participants differed significantly from the rest of the RCT sample were current marital status (63.6% of IDI participants were married compared to 84.6% of non-IDI participants, *p* = 0.011) and number of years since last HIV test [1.6 mean and 1.0 (0.3–2.3) median years among IDI participants compared to 1.0 mean and 0.5 (0.3–1.0) median years among non-IDI participants, *p* = 0.047].

**Table 1:** Descriptive characteristics of participants in the HIVST RCT and of those choosing not to test, Nakuru County, Kenya, 2015.

**Characteristic**	**No IDI (*****N*** = **281)**		**IDI (N** = **24)**		**Statistical significance of differences**
	***n***	**%**	**n**	**%**	***p*–value**
Age in years					0.676
Mean	37.0	37.3	
Median (IQR)	36.0 (32.0–41.0)	36.5 (28.0–44.5)	
Age group, years					0.117
≤24	5	1.8	0	0.0	
25–29	45	16.0	8	33.3	
30–39	138	49.1	8	33.3	
40–49	69	24.6	4	16.7	
50+	24	8.5	4	16.7	
High school graduate					0.851
No	181	64.4	15	62.5	
Yes	100	35.6	9	37.5	
Personal income from trucking (KSh)				0.513
8,000–15,999	13	4.9	2	9.1	
16,000–23,999	61	22.9	4	18.2	
24,000–55,000	192	72.2	16	72.7	
Years worked as truck driver				0.210
Mean	8.8	7.4	
Median (IQR)	6.9 (4.0–11.0)	5.0 (3.0–10.0)	
Currently married					0.011
No	43	15.4	8	36.4	
Yes	237	84.6	14	63.6	
Ever tested for HIV					0.239
No	25	8.9	0	0.0	
Yes	256	91.1	24	100.0	
Years since last HIV test					0.047
Mean	1.0		1.6		
Median (IQR)	0.5 (0.3–1.0)	1.0 (0.3–2.3)	

HIVST, HIV Self–testing; RCT, Randomized Controlled Trial; IDI, In–depth interview; KSh, Kenyan Shillings.

Chi–square tests were used for categorical variables, Fisher's exact tests where at least one cell was <5, and Wilcoxon rank–sum (Mann–Whitney) tests were used for continuous variables.

### Themes about preferences for type of HIVST biospecimen

There were mixed opinions about the benefits and challenges of the oral and blood-based finger prick, but slightly more participants indicated they preferred the blood-based test. Of the 14 participants who answered the question about preferred biological sample for HIVST, 9 preferred blood-based over oral, but 5 participants preferred oral HIVST. By study arm, a higher proportion of those who answered this question in the Choice arm (four of five participants) than in the SOC arm (five of nine participants) preferred blood-based HIVST.

**Figure 1:**
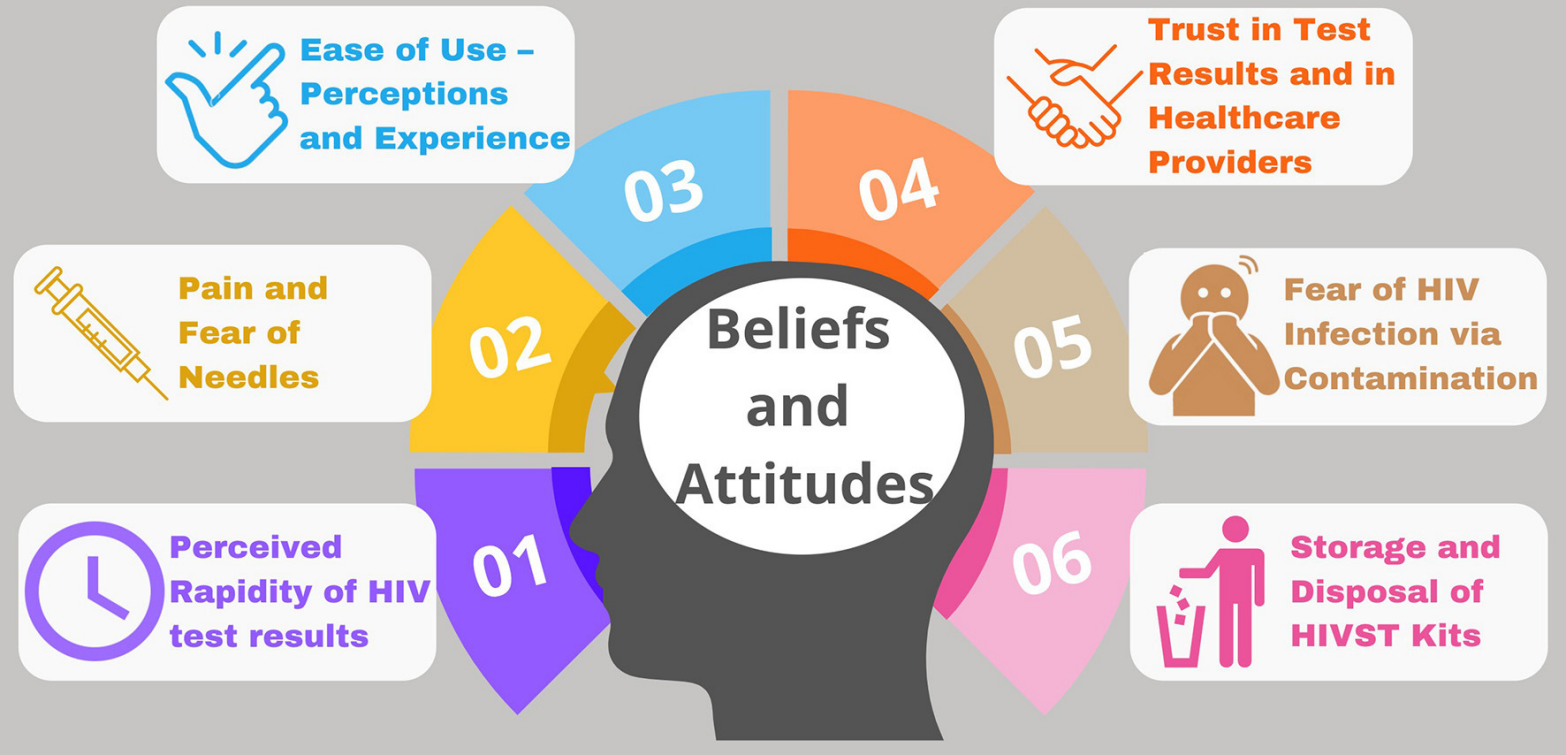
Attitudes, beliefs, and preferences—key themes.

Six key themes that described male truck drivers' beliefs and attitudes about oral and blood-based finger prick HIVST emerged. As shown in Figure 1, these included (1) Perceived rapidity of return of HIV test results. (2) Pain and fear associated with finger prick. (3) Ease of use. (4) Trust in test results (related to both provider support and to the test's ability to detect HIV). (5) Fear of infection by contamination; and (6) Testing location, storage, and disposal of used HIVST kits.

#### Perceived rapidity of return of test results

Even though both the facility-based provider-administered rapid blood-based test and oral self-test both only take 20 min for return of results and, in fact, there is an instant blood-based HIVST (Insti) but no instant version of an oral HIVST, participants had the misconception that the waiting time for receiving oral HIVST results would be shorter than that for a blood-based self-test and therefore would be more convenient and could reduce psychological stress.

*Because you get to know your results in 20 min…when one is testing, he gets very anxious to know the answer – to know you have or you don't have that disease, so it will make your heart calm down because the results* are fast. (Participant 5145, age 43, declined testing at baseline, Choice arm)

*Because I think it is much faster than the other one..*. (Participant 5063, age 28, declined testing at baseline, SOC arm)

#### Pain and fear associated with finger prick

Participants were concerned about the acceptability of the blood-based finger prick self-test, especially the combination of pain associated with pricking and potential for a positive test result.

…* there is that pain of pricking yourself and then you get another pain if you find you are not ok*. (Participant 5145, age 43, declined testing at baseline, Choice arm)

Some wondered whether people had the emotional disposition to prick themselves. This was a strong motivator for preferring oral HIVST.

*The one for pricking myself, that I can't* [do]. *Don't even talk about that one because I cannot see myself taking a needle and pricking myself. That is not possible. I am a coward… Maybe if someone holds my hand, that's when it will work*. (Participant 8091, age 41, declined testing at follow-up, Choice arm)

*There is no pain in the oral method, but the one for pricking hurts because of the pricking. That's why I only recommend* [the] *oral one. That is the best*…. *By the way I prefer that oral one because there is no pricking yourself then blood comes out. Some people fear being pricked…*. (Participant 9084, age 31, declined testing at follow-up, Choice arm)

[Finger prick test] *will be a bit hard unless one has a strong heart*…. *If it's that one for the mouth, I can really advertise it to my family and partner because I will tell them that they can test themselves and it doesn't have pain, you just brush it in your mouth and then put it here it shows you this, if the line goes there you have it and the other way round....They will think its ok because it's easy and not painful since if you have to prick yourself and take your blood and test, that could be a painful process*. (Participant 4153, age 46, declined testing at baseline, SOC arm)

#### Ease of use

Simplicity of use was another feature that predisposed participants to have positive attitudes about the oral HIVST. Sample collection was seen as being straightforward, not complex, and not dependent on a healthcare provider.

*That one doesn't have complications you can even self-test right now even when you are with your partner you can test her and see her status right then*. (Participant 8018, age 50, declined testing at follow-up, Choice arm)

…*you just put under your teeth so it's the simplest and the best way to test HIV*… *and very easy to use without need to have a healthcare provider around.…* (Participant 5145, age 43, declined testing at baseline, Choice arm)

However, some participants disagreed and believed that the blood-based finger prick test was easier to self-administer than the oral test. In the words of one participant,

*Because it will be easier to use than the others…. Getting blood samples from the fingers is easy*. (Participant 5011, age 28, declined testing at baseline, SOC arm)

#### Trust in test results

Of the nine participants who preferred blood-based HIVST, six reported that they favored it because they were more confident in its results. This trust was attributed to providers' apparent support for blood-based HIVST and the perceived advantage of blood-based HIVST's ability to detect HIV over that of oral HIVST.

#### Provider support

Trust was an issue noted with regard to both oral and blood-based HIVST. Some participants mentioned they trusted the efficacy of the oral self-test because it was supported and endorsed by healthcare providers.

*But you get confident because you know it must be working correct because the healthcare workers already approved it and they have a good reason for doing that provided you ensure you keep checking after three months*. (Participant 5145, age 43, declined testing at baseline, Choice arm)

*You will trust it because it is the doctors who invented it* (Participant 5135, age 26, declined testing at baseline, SOC arm)

Participants noted that the presence of a provider during the reading of test results would help to allay fears about oral HIVST results. This was especially important for participants who lacked confidence in conducting the test or interpreting test results and felt more secure with the presence of or assistance from a health care provider. They reasoned that providers are trained, know how to collect the sample, and therefore you would “*get your real status, not guess work.”* (Participant 5024, age 48, declined testing at baseline, SOC arm)

*Before getting tested the provider would have already explained how to interpret the result. So it is easy for you to read provided you have been told if it is, if it goes like this, it is like that…*. (Participant 5011, age 28, declined testing at baseline, SOC arm)

Confidence in using the oral HIVST kit was further bolstered by access to trustworthy information about how it works. Training in how to conduct the test and interpret the results was also perceived to enhance participants' confidence in using the oral test.

*Yes I trust...Because I will have already used it and gotten proper information about how it works so I will have no doubts about the results*. (Participant 5145, age 43, declined testing at baseline, Choice arm),

*I shall just recommend because if it's* [oral HIVST kit] *the one on the ground for use. I will have to explain to them and we know how to use it. You know once we have gone to a health facility, they will explain to us how to use the kit and how to read the result so that the person being tested can feel satisfied*. (Participant 5011, age 28, declined testing at baseline, SOC arm)

#### Test's ability to detect HIV

Understanding that HIV can be detected in oral fluids was given as a rationale for participants' trust in the oral HIVST results. One participant who mistakenly reasoned that the virus could be transmitted mouth-to-mouth because HIV was present in body fluids believed that the virus could easily be detected by swabbing the gums.

*Yes, I would trust. Because the fluids I have used are mine and everything is coming from me and nothing is external and because HIV can be transmitted from the mouth to another person through the fluids, it means those fluids carry the virus*…. *You know out of the education we have received, I know that HIV can be transmitted from bodily fluids which I think includes fluids that come out of the gums. Then I think even the oral one can also be a good way of testing*. (Participant 5021, age 32, declined testing at baseline, SOC arm)

However, there also was skepticism about the accuracy of oral HIVST to detect HIV or that oral fluid results are “guess work,” which dampened trust in this type of self-testing. Also, lack of familiarity with the oral fluid test undermined trust in its results. Participants noted that people were accustomed to the blood test.

*That one* [oral test] *I cannot do that one. You know I was told the method that is known is the blood test. This oral one you know….I cannot trust them* [results of oral test]. *That one I can't because there is no blood there….these oral tests you don't know* [if] *its saliva or what they now tell you to go place in your mouth. You could have even something else in the mouth and then it brings the bad results you had not expected. That one I don't trust*. (Participant 9067, age 38, declined testing at follow-up, Choice arm)

One participant made an analogy between car diesel and blood to explain why he refused the oral HIVST and preferred blood-based HIVST, which highlighted his lack of trust in the oral fluid self-test.

…*it's the blood that carries. The blood is the one that has the disease you know like in a car diesel, the diesel is the blood of the car, isn't it so. Do you think if the diesel has water or it is bad, can the car move? Will you go and test the water in the car because the water is the one causing the car not to start, or will you say that no, let me wash the engine and the oil remains in the car. Do you think that will do? It cannot work so it's better the blood than the one* [oral HIVST] *you are telling me*. (Participant 8037, age 38, declined testing at follow-up, Choice arm)

Longstanding messaging that HIV transmission *via* saliva is extremely low or non-existent sowed doubt that HIV could be detected in oral fluid.

*You mean the one to use in the gum? That one might not give results...Because many people say that saliva does not transfer any disease*…. (Participant 5024, age 48, declined testing at baseline, SOC arm)

Other participants believed that the virus can only be detected in blood, “it actually reveals if you are HIV-infected,” “it is more reliable,” “it gives the real results,” concluding the blood-based HIVST is better than the oral HIVST. The notion that blood flows throughout the entire body provided greater reassurance that HIV could be detected in the blood compared to in oral fluid.

*I won't have so much confidence. Because the way I know it, the virus is in the blood. So someone will lose faith when testing orally. He will decide to have his blood tested*.…*I would most likely recommend the blood test…because it is more reliable. Because you know blood circulates in the whole body, not like the saliva, and therefore is more reliable than saliva....They would feel good because everyone is able to see their own results*. (Participant 4071, age 28, SOC arm)

*Because blood is everything and if the blood is found to be infected, then that's it*. (Participant 5135, age 26, declined testing at baseline, SOC arm)

*The blood test is good because if you are infected it goes to the blood; any disease goes into the blood. You remove blood, it is tested. That is when you find it….that I think is better*. (Participant 9012, age 31, declined testing at follow-up, Choice arm)

Lack of familiarity and hence understanding about how the oral HIVST works made some participants question the accuracy of oral fluid test results.

*I have never used the oral self-test so I don't understand how it works and gives results especially that it doesn't use blood to test for HIV virus*. (Participant 4154, age 56, declined testing at baseline, Choice arm)

*I cannot believe the results of this kind of* [oral] *test. Because as per the information I have received, it is the one that uses blood that…gives correct results. I have never heard about the oral kit*. (Participant 5119, age 25, declined testing at baseline, SOC arm)

Regardless of whether participants held positive or negative views about the oral HIVST, they highlighted the need to have a confirmatory facility-based HIV finger prick blood test if their HIVST result was reactive.

#### Fear of infection by contamination

Concerns about the potential for infection due to contaminated blood-based HIVST devices, e.g., acquiring other diseases due to an unclean lancet, motivated preference for the oral HIVST among some.

…*sometimes I feel that the lancet used to prick may infect me with other diseases…*.(Participant 5021, age 32, declined testing at baseline, SOC arm)

*You know I am afraid I may prick myself and maybe the lancet I have used has rust or is dirty which will be a challenge for me to test myself*. (Participant 4154, age 56, declined testing at baseline, Choice arm)

Some were concerned about re-use of the blood-based test kit, e.g., giving the test kit they used to a friend and infecting that person.

… *because when I use it alone, I can take the needle and you know just a prick will get blood now I can take it to someone else to use it*. (Participant 4153, age 46, declined testing at baseline, SOC arm)

…*the bad thing is that they can use one needle for two people*. (Participant 5029, Age 28, declined testing at baseline, SOC arm)

*You prick yourself and someone else uses the same which will be wrong*. (Participant 9012, age 31, declined testing at follow-up, Choice arm)

#### Testing location, storage, and disposal of HIVST kits

Participants indicated a number of places that they thought were fitting to test themselves, including at truck stops, in their truck, at home, or in the clinic at the truck stop.

*Now I can test from the house or anywhere else, in the car, in the house, at the clinic, here at your center*. (Participant 4153, age 46, declined testing at baseline, SOC arm)

*Like in my truck when am traveling but very relaxed or even in the guest house*. (Participant 5145, age 43, declined testing at baseline, Choice arm)

Relatedly, concerns about insecure storage and unsafe disposal of blood-based HIVST kits and the potential for causing harm to others were expressed.

*The problem with this is that you may find people.…poor disposal where children can find them and start playing with them*. (Participant 5024, age 48, declined testing at baseline, SOC arm)

*They can leave them carelessly and kids play with them*. (Participant 5029, age 28, declined testing at baseline, SOC arm)

## Discussion

Our qualitative interviews provided a new lens to explore preferences for type of HIVST biospecimen and the beliefs behind those preferences among a sub-sample of truck drivers who declined HIV testing when offered in the context of a research study evaluating the impact of adding oral HIVST as an option in a clinic system. It also afforded insights into the misconceptions and mistrust driving some of these preferences that could be addressed with appropriate messaging.

### Variation in preference

We found variation in preference for an oral fluid vs. blood-based HIVST, but preference for a blood-based self-test appeared to be stronger among those in the Choice arm who were offered oral HIVST. This is likely because most of those who preferred oral HIVST accepted testing when that is what was offered and thus were excluded from the IDI sample. However, we cannot characterize this as a definitively clear pattern due to the small sample size.

It is possible that those refusing testing in the Choice arm might have self-tested had we offered a blood-based self-test, rather than only an oral HIVST option, thus increasing testing uptake.

That we found differing preferences regarding the biological specimen used for HIVST is similar to what we found in the Discrete Choice Experiment (DCE) embedded in the same RCT, that is – no clear preference for oral vs. blood HIVST on average, but those who had never tested preferred oral fluid over a finger-prick test, whereas those who had tested before were indifferent (40). A DCE study among young women and men aged 16–25 in Malawi and Zimbabwe also did not identify any significant preference for specimen collection method; focus group findings in both countries noted benefits of both oral fluid and blood-based HIVST (41). Other studies have discerned variation in specimen preference; as reported in a recent systematic review of 63 HIVST studies among men in SSA conducted between January 2010 and June 2020, both oral fluid and blood-based HIVST were found to be highly acceptable (42).

In contrast, some preferences for test type were found in several of the limited studies focusing on both oral fluid and blood-based testing. Studies among MSM in South Africa (16), adolescents in Thailand (43), heterosexual men in Singapore (44), and MSM in the UK (45) found that participants preferred blood-based HIVST on average for reasons such as rapidity of results and trust in the perceived accuracy of results. However, within these studies, some participants cited benefits of oral fluid HIVST such as ease of use and ability to avoid fear of needles.

Other studies found a stronger preference for oral fluid than blood-based HIVST; participants such as MSM in Australia (46), adults in the Democratic of the Congo (47), and youth in Nigeria (48) preferred oral fluid more on average. However, within all these studies, preference for one test over the other was never ubiquitous. Participants varied in their preferences, and preferences were sometimes correlated with other factors, like education and frequency of testing (47).

Overall, these studies suggest there is no clear winner regarding HIVST biospecimen preferences. This is consistent with our study finding that some preferred blood and others oral HIVST. One explanation for the lack of consensus is that preference for blood-based HIVST may be influenced by previous experience testing for HIV with a provider, which traditionally has been by finger prick. Clients who have a history of HIV testing may be accustomed to and comfortable with the collection of this type of specimen compared to lack of familiarity with the oral fluid HIV test. For example, a study in Tanzania found that oral swabs were the least preferred method for obtaining the sample for the HIV test among 70% of participants. However, men who had never tested were more likely to prefer oral swabs compared to those who had tested previously (49). Another study found that preferences reflected whichever type of test kit they used recently (50), signifying that familiarity with the test influences preferences. As a result, it may be that most individuals would prefer access to both types of testing, if possible, as found in a study in the Democratic Republic of the Congo (47).

Our findings confirm what emerges from the literature: some people prefer oral testing whereas others prefer blood-based tests. Only by offering choice will there be an option to suit more people (40), potentially increasing HIVST uptake. This “choices” strategy has proven useful in the dissemination of other biomedical technologies like contraception (51). Thus, an optimal HIV testing program might be one that offers a variety of options. Offering multiple HIV testing choices (i.e., both provider- and self-administered oral and blood tests) has not been sufficiently tested, so we do not know the optimal number or combination of testing options needed to maximize uptake. Most research on HIVST has focused on the oral-swab test, with little information about the acceptability, uptake, and ease of use of blood-based HIVST kits compared to oral HIVST. This is not due to the lack of availability of blood-based HIVST. As of August 2022, six HIVST have been prequalified by the World Health Organization (5 using whole blood and one using oral fluid) as of 2022: Wondfo, Check Now, Sure Check, Mylan, Insti, and OraQuick (52, 53). Ensuring availability of different HIVST kits on the market may help increase testing demand. In the current environment of slow but increasing number of blood-based and oral HIV testing strategies, offering choices in HIVST strategies may help to close the gap between HIV testing and treatment.

Lack of diversification of HIVST products offered in studies limits our understanding of HIVST test preferences and their effects on uptake. Researchers should use caution in making any generalizations about HIVST uptake, acceptability, or preferences for test characteristics based on studies that offer only one type of HIVST (28). For example, a study of young MSM in the US randomly assigned participants to free home-based, oral fluid rapid HIVST, free home-based mail-in blood sample collection HIVST, or rapid or conventional testing at a health facility or community organization of their choice, and found that oral fluid rapid HIVST was preferred to home-based mail-in blood sample collection (54). While they found that oral HIVST did not result in greater testing compared to rapid or standard testing at facilities or community organization sites, their findings are only applicable to oral HIVST and cannot be extrapolated to HIVST in general. This problem is also apparent in nearly all HIVST RCTs, for example, in studies with female sex workers in Zambia and Uganda which compared two intervention arms that provided direct delivery of HIVST kits or a peer-delivered coupon to pick up a test kit at a health facility, with referral to standard of care provider-administered HIV testing arm (55–57). HIV testing uptake was higher in the intervention arms than the SOC arm, but we cannot determine whether the higher uptake among those offered HIVST over SOC was due to a preference for self-testing or a preference for an oral HIV test.

Although we cannot definitively answer the question about whether the type of specimen used in HIVST is a primary barrier or facilitator of testing, we found that some participants did not trust oral HIVST due to its perceived lower accuracy compared to blood-based testing. This stems from the incorrect belief that HIV cannot be detected reliably from “saliva” and may be related to longstanding prevention messages about HIV acquisition and transmission that stress that there is little to no risk of HIV transmission though oral sex (58, 59), kissing and sharing utensils. By logical extension then, the ability to detect the virus *via* oral fluid HIVST must be low. The Fieldworkers in our main RCT echoed this sentiment of skepticism from participants and found it difficult to explain how the oral test detected HIV in oral fluid when HIV cannot be transmitted orally, especially in Kiswahili.

Results from other studies suggest that perceived inaccuracy of the oral test is not specific to our study population. Fear of inaccurate results with oral HIVST and distrust of an oral fluid screening test have also been reported in a qualitative study of 35 pregnant women presenting for antenatal services in a rural Indian hospital who self-tested with the OraQuick^®^ kit (60). In eThekwini (Durban), South Africa, a qualitative study of 20 people presenting at primary health care centers, some indicated they would not self-test because they would not trust the oral test results (17). This uncertainty about the accuracy of oral HIVST results has also been seen in studies of MSM and people at elevated HIV risk. In a 2021 study in Bangkok, Thailand, among 87 adolescents and young adults in which participants were offered a choice to use either the blood-based HIVST (INSTI^®^) or the oral-fluid based HIVST (OraQuick^®^), two-thirds (65.6%) preferred blood-based HIVST (43). Rapid return of results and higher accuracy were given as reasons for preferring blood-based HIVST (77.2 and 66.7%, respectively). In a cross-sectional study that compared the practicability, accuracy of and preference for the blood-based HIV self-test *vs*. the oral-fluid-based HIV self-test among 528 participants at risk for HIV in the Democratic Republic of the Congo, 23% reported they did not trust the results of the oral fluid test, whereas 25% feared using the lancet of the blood-based test. Participants in another study, which included female sex workers and MSM in China, saw many advantages of oral fluid HIVST, yet among the female sex workers, more than half doubted the accuracy of oral test results (61). In a qualitative study of 30 young black MSM and transgender women in the New York City area, some participants' attributed a strong preference for blood-based HIV tests to their greater confidence in its accuracy and fewer false negatives compared to oral fluid tests (62).

Two key focus areas for programmatic implementation emerge from the thematic analysis. Both relate broadly to knowledge about HIV transmission and testing options: (1) misconceptions and lack of understanding; and (2) gaps in knowledge where people are likely to require additional education and information about self-test kits and how they work. These findings also point to topics for future exploration in research about HIVST that is needed to close the gap between clinical trials and widespread acceptance of HIVST in real-world settings.

### Misconceptions and lack of understanding

A number of misconceptions are concerning and should be addressed by interventions to improve HIV-related knowledge and trust in healthcare providers. Participants had misunderstandings about how HIV antibodies could be detected in oral fluid. Oral fluid, i.e., oral mucosal transudate, is rich in antibodies that originate from the plasma and are transferred across the oral mucosa and gums (63). Furthermore, participants did not know that the body reacts to HIV infection by creating HIV antibodies, which can be detected in oral fluid even though there is little or no actual virus in oral fluids. These issues should be addressed through education. Furthermore, the belief that the lancet used in blood-based HIVST kits might be used multiple times and lead to HIV infection indicates misunderstanding about how the kits can be used, which may mirror the mistrust of HIV testing by providers described by one participant. This also needs to be tackled through messaging that targets misinformation and fear.

Another misconception identified in our study that may inform preferences was that oral HIVST would return results more quickly than blood-based HIVST. Participants in our study mistakenly believed that the speed of return of HIV test results was more rapid for the oral fluid than the blood-based self-test, possibly due to participants' experience waiting for long periods in a health facility for their blood-based provider-initiated test which might be more due to the clinic being busy than the time for test results. Quick return of results was also one of the reasons for preferring oral fluid over blood-based HIVST identified by a qualitative study conducted with MSM and transgender individuals in Pune, India (64). However, another study in Kenya noted the opposite—that results from the blood-based test were thought to be returned more quickly, e.g., “It is very efficient for me I go with time... I don't need to wait for like the oral one 40 min. I can get my results instantly” (65). In actuality, the INSTI^®^ blood-based HIVST has a shorter turnaround time (1 min upon completion of the procedure) than the OraQuick^®^ oral fluid test (20 min), although the testing procedures for INSTI involve more steps than other HIVST kits so the testing process may actually take more time (28, 66, 67). However, the time for return of results with the Mylan HIV self-test (Atomo Diagnostics) is 15 min, similar to the waiting time for OraQuick^®^ test results (68).

Since the beliefs about HIV detection accuracy, risk from the test and rapidity of return of results differed according to biospecimen, they could influence the impact of offering HIVST on HIV testing rates if only one type of HIVST is offered. Thus, accurate messaging that targets some of these misconceptions about how the various oral fluid and blood-based self-testing kits work is an essential component of the rollout of HIVST programs. The array of beliefs, attitudes, and misconceptions about oral fluid and blood-based HIVST highlights the need for exploratory research. HIV self-testers need to understand that HIV can be detected by swabbing the mouth, even though risk of oral HIV transmission from oral fluids is extremely low or non-existent—a message that has been promoted for decades. Messaging that HIV is not easily transmitted orally but that infection can be identified because biomarkers of HIV infection, specifically antibodies generated by the infected individual in response to the infection, are present in oral fluid, should be disseminated. The details surrounding these concepts are not easy to explain and educational materials that simplify and clearly elucidate these important concepts, alongside strategies to mitigate potential users' objections and build trust in both types of tests, need to be taken up by healthcare providers. In addition, details about how each HIVST kit works could allay fears about re-use of lancets and misinformation about the time required to complete the test. Trust and understanding are key to building demand for HIVST.

Understanding the beliefs that underlie preferences can help programs address client predilections. For example, an mHealth tool used in a Cape Town, South Africa HIV testing program aims to provide information to people using oral HIVST to address misconceptions like the perceived contradiction about HIV transmission *via* saliva (69). This will become increasingly important as many of the blood-based self-tests in the pipeline come on the market, some of which are already available in Kenya (65) as well as in other countries. As a case in point, a qualitative study of adolescents and adults and providers from private-sector pharmacies and clinics in Nairobi and Mombasa conducted by Population Services International found reported variability in preferences for types of HIVST kits. The value of the blood-based finger prick HIVST was directly linked to beliefs about blood and diseases: e.g., “I would prefer the blood based because blood is a component that is more direct to diseases than the saliva,” as noted in other studies (65, 70), including this one.

### Need for additional education and information about HIVST

This variation in preferences suggests that by offering HIVST choices we may be able to increase HIV testing uptake when HIVST is made available. However, there were some valid concerns expressed, such as safe disposal of blood HIVST so others do not come into contact with used lancets. Participants noted that HIVST kits provided flexibility in where they could test. However, concerns about storage and disposal of test kits were voiced only in relation to the blood test, especially the safety of disposing the lancet. The interviews did not explicitly ask about storage and disposal; rather this issue emerged organically in the interviews. We did not find reports about storage of kits or potential exposure of sharp objects in the literature we reviewed. Storage and disposal of HIVST kits would be especially relevant for many at-risk and mobile populations like truck drivers and sex workers who might find it challenging to store kits prior to using them and dispose of these products appropriately and discretely after use. As pointed out by participants, disposing of blood-based test kits is especially important given that it may result in finger-prick incidents for others who play (children) or dispose of them. Potential users may also be concerned about finding a private storage space for HIVST kits to preclude inadvertently disclosing their testing behavior to others, although this did not come-up in our interviews and should be explored in future research.

### Study strengths and limitations

HIVST is a key tool in helping individuals to monitor their HIV status and mitigate transmission of the virus to others, especially when concerned about potential stigmatization. It also is an important component of a self-care package of sexual and reproductive health services, which aims to allow users to manage their health without necessarily having to access health facilities. Our study addressed a gap in evidence by providing more specifics about a key population's preferences for type of biospecimen in HIVST kits and disentangles some of the conflation between self-testing and oral testing which appears to dominate previous research and some of the key messaging around HIVST. During 6-month follow-up, our quantitative assessment indicated that oral swab preference was lower among those in the intervention, who had seen and had the opportunity to use the oral HIVST than those in the SOC arm (71). Interviewing men who declined HIV self-tests in the RCT is an informative implementation science approach for understanding the mechanisms of why and how the parent RCT did or did not observe an effect. It highlights the importance of nesting qualitative sub-studies within RCTs as a “best practice” to understand an intervention's effect on the outcome of interest.

Notwithstanding the study's strengths in describing HIVST biospecimen preferences and the beliefs behind those preferences, several limitations must be noted. First, social desirability bias may have played a role in how people answered the questions. For example, those in the Choice arm who refused testing might have felt that they needed to express a preference for a blood-based HIVST to explain their decision not to test when an oral HIVT was provided. However, that the IDIs were administered a few months after the baseline or follow-up testing opportunities might make this less likely. Second, the 24 truck drivers included in this component of the study may not have represented the whole range of views among participants who refused HIV testing. In addition, the sample size made it difficult to discern definitive patterns in preferences for type of HIVST biospecimen. Third, IDI participants were a subset of study participants who declined HIV testing at some point in the study and therefore their views may differ from those in the study who were ineligible for or eligible for but did not participate in the IDI component of the study. Unlike quantitative studies, qualitative studies are not designed to be representative such that you can generalize results to the population but rather to explore concepts pertinent to specific subgroups to understand their behavior and choices. We note that men in both the SOC and Choice arms were not shown a blood-based HIVST kit, although they were familiar with provider-administered finger prick testing used in the clinics, so their preferences were not based on experience with all the products being discussed. Finally, the data were collected in 2015–2016, prior to Kenya's roll-out of HIVST in 2017 and may reflect lack of knowledge about and the novelty of HIVST at that time with this form of HIV testing not being fully understood by participants. Beliefs and preferences may have changed over time as HIVST becomes more available and established. However, blood-based HIVST kits are still not widely available in the public sector and non-governmental clinics; thus, users of these clinics do not have the option to choose the type of HIVST biospecimen.

### Implications for future research

**Figure 2:**
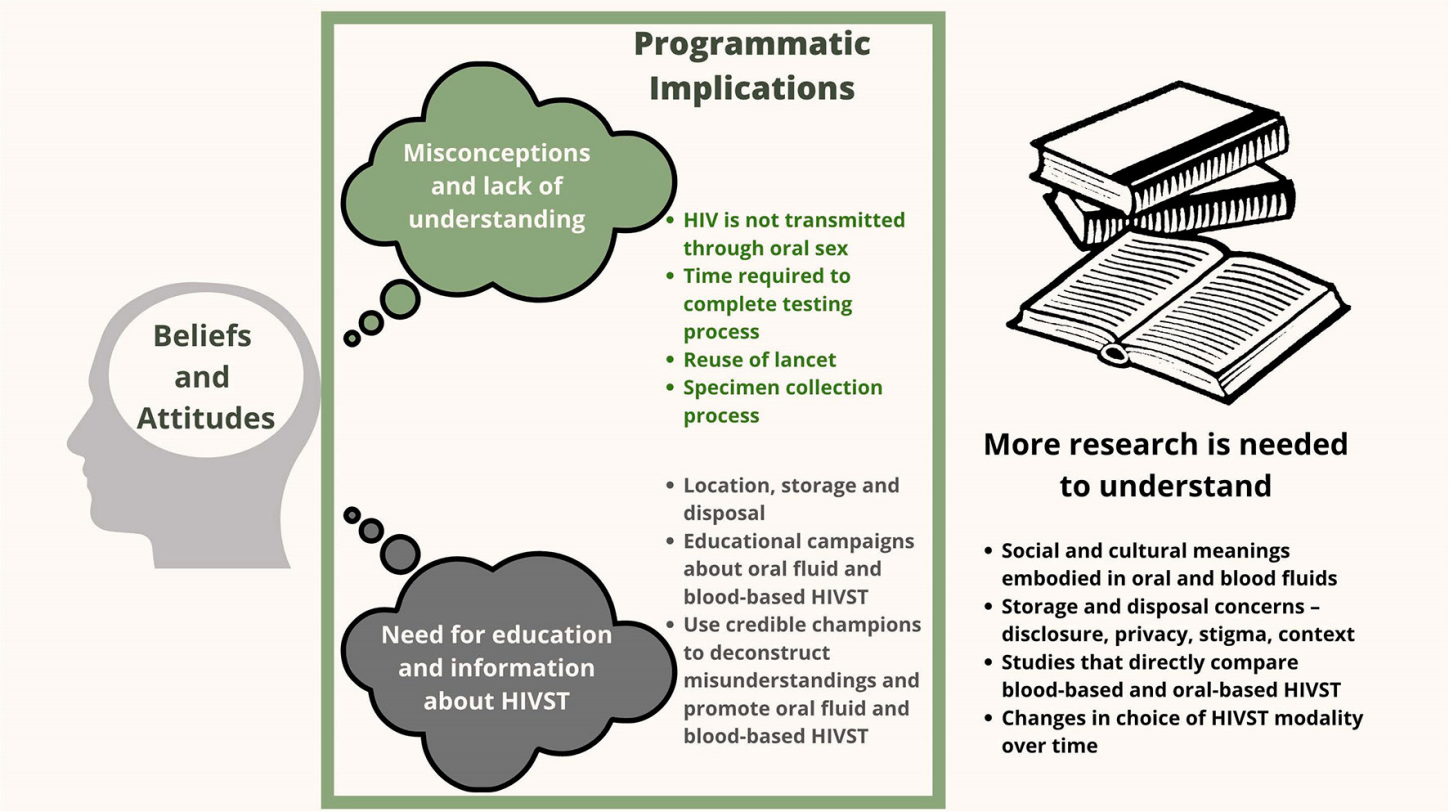
Programmatic implications and the need for future research.

Figure 2 presents programmatic and research implications of study findings. Offering broader choices for HIVST could have a greater impact on testing uptake, but more research is needed to address misconceptions so people make appropriately informed choices, and then understand what proportion of individuals would prefer oral vs. or blood-based HIVST kits once they have accurate understanding and why. Thus, HIVST programs might include information and trust-building campaigns to maximize program impact. In addition, although not addressed in our study, some literature suggests that bodily fluids like saliva and blood may be associated with certain symbols or cultural meanings, both positive and negative, and these perceptions may vary by social and cultural context (72–75). More research is needed to understand the beliefs that drive HIVST preferences and whether these preferences change over time as experience with HIVST increases. Future qualitative research could help HIVST programs to understand what education and counseling should accompany an HIVST program, and what dissemination channels and messengers are most effective in promoting HIVST choices for specific populations.

## Conclusions

This study highlights the importance of aligning individuals' HIVST preferences with the supply of HIVST kits. To cater to the preferences of a diverse population, a combination of testing modalities is needed to achieve high coverage. As with other biomedical technologies, one size is unlikely to fit all. Our qualitative inquiry provides some nuanced insights into how truck drivers in Kenya interpret an innovative HIV biomedical technology, in this case, HIVST products, and why they might choose not to use specific HIVST products and the reasons behind those decisions. The study underscores the diversity in preferences and beliefs and thus the importance of offering choices in an HIVST program. In addition, some areas of concern included misinformation and mistrust that need to be addressed to maximize the potential of this new biomedical technology. HIVST research and new product development are ongoing. Research suggests that the pace of endorsing and adopting a new biomedical innovation as a mainstream strategy can be slow (76–78), taking more than a decade for new products to be integrated into routine clinical practice (79). We have witnessed the slow and inconsistent uptake of other new HIV and STI prevention technologies, as reflected in experience with daily oral pre-exposure prophylaxis and likely with the cabotegravir 2-month injectable and the dapivirine vaginal ring. Mixed-methods research can elucidate why uptake of a new biomedical technology might be slow or plateau, thus informing dissemination and implementation science strategies to increase uptake over time. In the case of HIVST, blood and oral tests are available which gives people choices. Understanding the preferences, beliefs, and attitudes of target populations and ensuring multiple options to meet the diversity of preferences, coupled with messaging to educate and build trust among potential users, are key to informing the design of appropriate intervention programs to maximize HIV testing rates, especially for vulnerable populations.

## Data availability statement

The datasets presented in this study can be found in online repositories. The names of the repository/repositories and accession number(s) can be found below Harvard Dataverse repository: https://dataverse.harvard.edu/dataset.xhtml?persistentId=10.7910/DVN/8GVXJY. The qualitative data are available upon request and approval from the study investigators.

## Ethics statement

The studies involving human participants were reviewed and approved by City University of New York Institutional Review Board, the Kenya Medical Research Institute Ethics Committee, and the University of KwaZulu-Natal Biomedical Research Ethics Committee. The patients/participants provided their written or oral informed consent to participate in this study.

## Author contributions

EK led the conception and design of the study. JM, SC, and MR conducted the data analysis. MR and SC coded the data. MR, SC, and AK participated in data interpretation. JM and AK drafted this manuscript, with EK, MR, GG, MS, EM, and KG contributing to manuscript development and revisions. All authors read and approved the final manuscript.

## Funding

This study was supported by a grant from the International Initiative for Impact Evaluation (3IE, Grant #OPP106693, TW2.2.06 supplement, EK, Ph.D., Principal Investigator). EK was also supported by the Einstein-Rockefeller-CUNY Center for AIDS Research [P30-AI124414] which was supported by the following National Institutes of Health (NIH) Co-Funding and Participating Institutes and Centers: NIAID, NCI, NICHD, NHBL, NIDA, NIMH, NIA, FIC, and OAR. Support for JM also came from a center grant from the National Institute of Mental Health (NIMH) to the HIV Center for Clinical and Behavioral Studies at the New York State Psychiatric Institute and the Department of Psychiatry, Columbia University Irving Medical Center [P30-MH43520; Principal Investigator: Robert H. Remien, Ph.D.]. Support for AK came from the National Institute of Allergy and Infectious Diseases (NIAID) of the National Institutes of Health under Award Number [T32AI114398; Principal Investigator: Andrea A. Howard, MD, MS].

## Conflict of interest

The authors declare that the research was conducted in the absence of any commercial or financial relationships that could be construed as a potential conflict of interest.

## Publisher's note

All claims expressed in this article are solely those of the authors and do not necessarily represent those of their affiliated organizations, or those of the publisher, the editors and the reviewers. Any product that may be evaluated in this article, or claim that may be made by its manufacturer, is not guaranteed or endorsed by the publisher.
